# Facial palsy after administration of immune checkpoint inhibitors: case report, literature review and clinical care management

**DOI:** 10.3389/fimmu.2024.1375497

**Published:** 2024-03-22

**Authors:** Essia Mezni, Giovanni Corazza, Roxane Mari, Stephanie Coze, Nathalie Charrier, Brice Chanez, Anne Sophie Chretien, Philippe Rochigneux

**Affiliations:** ^1^ Medical Oncology Department, Paoli-Calmettes Institute, Marseille, France; ^2^ Neurology Department, Assitance Publique Hôpitaux de Marseille, Aix-Marseille University, Marseille, France; ^3^ Referral Centre for Neuromuscular Diseases and Amyotrophic Lateral Sclerosis (ALS), Hôpital La Timone, Marseille, France; ^4^ Radiology Department, Assitance Publique Hôpitaux de Marseille, Aix-Marseille University, Marseille, France; ^5^ Nuclear Medicine Department, Paoli-Calmettes Institut, Marseille, France; ^6^ Team Immunity and Cancer, Centre de Recherche en Canceírologie de Marseille (CRCM), Inserm, U1068, Centre national de la recherche scientifique (CNRS), Unité Mixte de Recherche 7258 (UMR7258), Paoli-Calmettes Institute, Aix-Marseille University, Marseille, France

**Keywords:** cerebral palsy, immune checkpoint inhibitors, immune toxicity, neurological toxicity, nivolumab, ipilimumab

## Abstract

Neurological immune-related adverse events (irAEs) due to immune checkpoint inhibitors (ICI) are rare complications of immunotherapy, particularly dreadful for patients and clinical teams. Indeed, neurological irAEs are potentially severe and their diagnosis require prompt recognition and treatment. Additionally, the spectrum of neurological irAEs is broad, affecting either neuromuscular junction, peripheral or central nervous system. Here, we described the case of a 55-year man with metastatic melanoma, facing a brutal right peripheral cerebral palsy after his third ipilimumab/nivolumab infusion. After the case presentation, we reviewed the literature about this rare complication of immunotherapy, and described its diagnosis work-up and clinical management.

## Introduction

Immune checkpoint inhibitors (ICI) reinvigorate patient’s own immune system to recognize and destroy tumor cells (1). These treatments, notably the CTLA-4 inhibitor ipilimumab (Ipi) and the PD-1 inhibitor nivolumab (Nivo), revolutionized the treatment of advanced solid tumors (2). Indeed, in metastatic melanoma, a first-line combination of Ipi and Nivo lead to an impressive 5-year overall survival of 52% (3). However, these efficient treatments have numerous immune-related adverse events (irAEs), with a broad spectrum of severity, that potentially affect every organ of the patient (4). If the onset of irAES are frequently associated with increased efficacy of ICI (5), their management in routine clinical practice remain challenging, as each of them requires specific diagnostic procedure and sometimes specific treatments (6).

Among every irAES, neurological irAES (N-irAES) are particularly difficult to manage, for several reasons: i) contrary to frequent irAES, like thyroid dysfunction or cutaneous toxicity, neurological complications might be life-threatening; ii) neurological complications are highly polymorphic as they might affect central nervous system (encephalitis, meningitis), peripheral nervous system (peripheral neuropathy, Guillain-Barre syndrome) or neuromuscular junction (myositis, myasthenic syndromes) (7); iii) the differential diagnoses are numerous (cancer progression, paraneoplastic syndrome, stroke…) and iv) the radiological and biological work-up necessary to diagnose N-irAES are frequently highly technologic (brain MRI, specific autoantibodies) or invasive (lumbar punction). Consequently, medical oncologists need clear knowledge to face neurological irAES and case descriptions are helpful in clinical practice.

Here, we report the case of a patient with metastatic melanoma experiencing a brutal facial palsy after three infusions of ipilimumab/nivolumab.

## Case presentation

**Figure 1:**
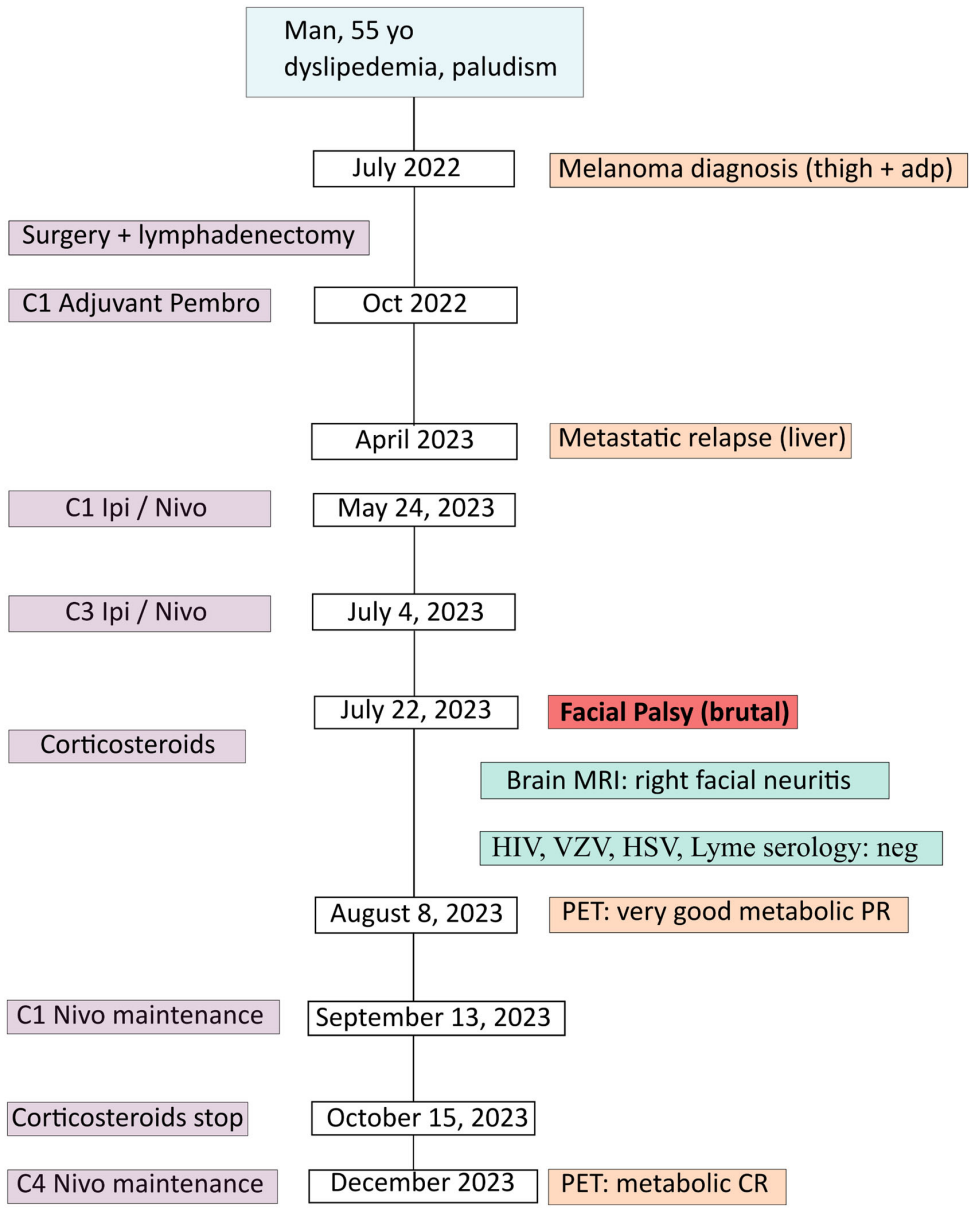
Patient’s timeline.

A 55 year-old man, ECOG-PS 0, with a medical history of dyslipidemia and non-severe malaria presented a right inguinal adenopathy, gradually increasing in volume, associated with a black skin lesion of the right thigh, surrounded with a perilesional vitiligo. In July 2022 (**Figure 1**: patient’s timeline), he consulted in our center (Paoli-Calmettes Institute, Cancer Center in Marseille, France) and beneficiated from a right inguinal lymph node biopsy (July 22, 2022). The histology revealed a malignant melanoma, PS100^+^, HMB 45^+^ and MelanA^+^. The PET-CT confirmed a hypermetabolic skin lesion with large right inguinal adenopathies, without distant metastasis and the cerebral MRI was normal. The next generation sequencing (in-house panel of 96 genes) revealed a BRAF mutation in exon 15, V600D (p.(Val600Asp), a punctual variation of TERT promotor (c.-146C>T) and focal homozygous deletions of genomic regions containing the loci CDKN2A and CDKN2B and TERT.

**Figure 2:**
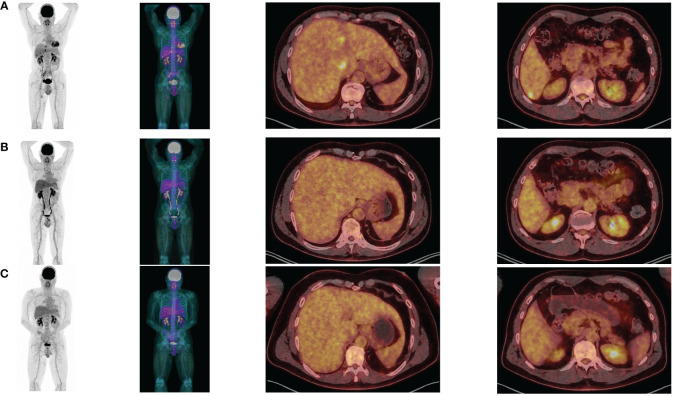
PET-CT at baseline **(A)**, after 3 Ipilimumab/Nivolumab combinations **(B)** and after 3 Nivolumab maintenance **(C)**.

In August 2022, the patient had surgical excision of the skin lesion and a right inguinal lymph node dissection. As recommended in tumor board, the patient started adjuvant PD-1 inhibitor (pembrolizumab 200 mg/3 weeks), with C1D1 in October 2, 2022. Unfortunately, in April 2023, after only 6 months of adjuvant pembrolizumab the patient relapsed with multiple metastatic liver lesions and multiple sub-diaphragmatic lymph nodes, particularly in the right iliopelvic region (PET-CT of April 27, 2023: **Figure 2A**). The new tumor board recommended either the inclusion in a clinical trial (investigating Ipi/Nivo +/- anti IL-8) or the Ipi/Nivo combination in routine clinic that the patient preferred. The first three infusions of Ipi/Nivo were well tolerated [C1: May 24, 2023 (Day 1)/C2: June 13, 2023/C3: July 04, 2023], with only a grade 1 dermatitis with pruritus.

**Figure 3:**
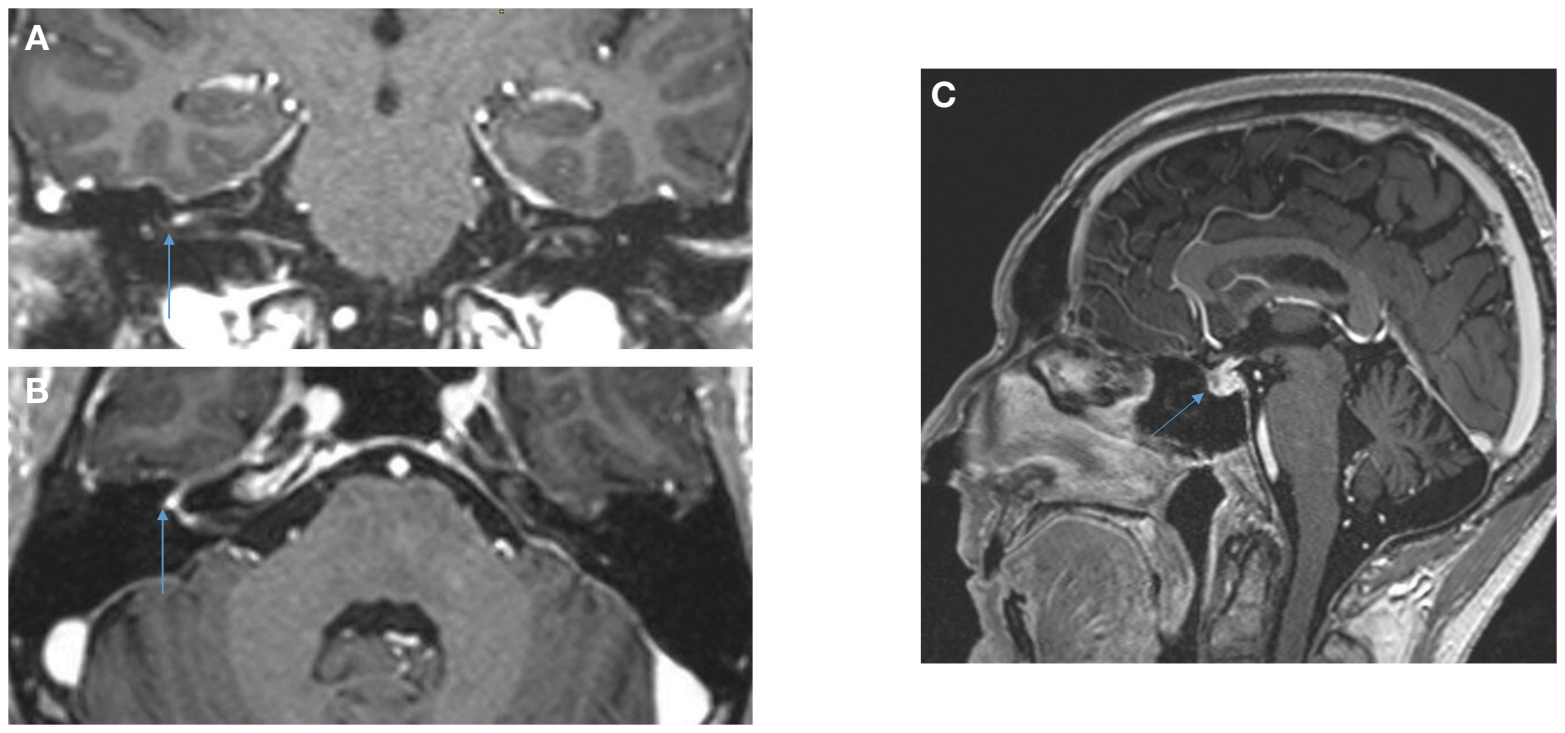
Cerebral MRI, Coronal **(A)** and axial **(B)** 3DT1MPRAGE sequences after gadolinium reconstruction showing contrast of the fundus of the internal auditory meatus and of the right geniculate ganglion attesting to right facial neuritis. Hypophisitis is shown in the sagittal sequence **(C)**.

However, on July 22, 2023 (Day 59), the patient presented with acute peripheral left facial palsy (grade 3 on CTCAE v5.0), with retro-auricular pain and numbness of the face. He consulted the emergency department in a local hospital. The clinical examination did not show any associated neurological signs (motor or sensitive deficit of the limb, cognitive impairment, additional cranial nerve injury) and the biological tests were normal. The cerebral CT scanner was normal. A diagnosis of Bell’s palsy was suspected: lumbar puncture was not performed and a treatment of oral prednisolone 1 mg/kg (80 mg) was started, together with ocular protection. The cerebral MRI in August 1, 2023 revealed no secondary localization, no signs suggestive of a stroke, but inflammation of the right facial nerve, suggestive of facial nerve neuritis (**Figure 3**). HIV, VZV, HSV and Lyme (*Borrelia burgdorferi*) serology were negative, glycemia and TSH were normal. Altogether, the concertation between oncologists and neurologists adopted the probable diagnosis of an immune-related facial palsy caused by ipilimumab/nivolumab combination. Consequently, we decided to stop ipilimumab/nivolumab combination.

The clinical evolution was favorable: after 10 days of prednisolone, the facial palsy improved and the patients began to close his upper right eyelid. The prednisolone was decreased by 10 mg every week (until 10 mg, then 5 mg and stop) and in September 2023, the patient did not present any sign of cerebral palsy, nor ocular complication. Interestingly, two other irAEs were observed: i) an hypophysitis revealed by the brain MRI of August 1, 2023 (diffusely enlarged pituitary gland, not shown) and a corticotropic deficiency (8 a.m. blood level cortisol: 2 nmol/l; blood level ACTH: 0.7 pmol/l); ii) a vitiligo around 20% of body surface starting in September 2023.

Concerning efficacy, the PET-CT after three infusions of Ipi/Nivo (August 9, 2023) revealed a very good metabolic partial response (liver metastases of segment I and IV, right external iliac adenopathy) and a complete response on other liver metastases and other sub-diaphragmatic lymph nodes (**Figure 2B**). We canceled the fourth and last ipilimumab/nivolumab combination and started the maintenance therapy by nivolumab monotherapy 480 mg q4w in September 13, 2023. PET-CT after three nivolumab maintenance (December 20, 2023) revealed a metabolic complete response in liver and lymph nodes (**Figure 2C**). At the date of redaction of this case report, the patient was ECOG-PS 0, maintained an intense professional activity without any clinical sequelae of the metastastic melanoma or the former facial palsy.

## Literature review and discussion

Here, we report the case of a patient presenting a facial palsy after 3 infusions of ipilimumab/nivolumab. As ICI-induced facial palsy might be challenging for oncologists, we will discuss the literature and the clinical management of this rare neurological irAE.

### ICI-induced facial palsy: literature review

The first case of ICI-induced facial palsy was reported in 2015 by Altman et al. (8). The physiopathological mechanism of this neurological adverse event remains unknown to date, even if an immune tolerance breakdown is the most probable (9). Although rare, facial palsy has been reported in the literature with different ICIs agents and combinations: ipilimumab (10) pembrolizumab (11), atezolizumab (12), nivolumab (13), ipilimumab plus nivolumab (14), ipilimumab plus pembrolizumab (10), tremelimumab plus durvalumab (15). The **Table 1** summarizes the main reported cases of facial palsy induced by ICIs reported in the literature. Interestingly, brain MRI was normal in approximately 50% of the cases and an additional irAE was observed in approximately 30% of the cases. In our patient, whereas ICI monotherapy by pembrolizumab was not associated with irAEs and let to tumor progression, Ipi/Nivo combination led to three irAEs (notably facial palsy) and a metabolic complete response at 7 months.

**Table 1:** Published cases of patients with checkpoint inhibitor-induced facial palsy (literature review).

Authors	Diagnosis	Gender	Age (years)	Treatment	Cycles before irAEs	Clinical presentation	MRI	Management	Duration of symptoms	outcome	ICIs discontinuation	Other immune toxicity
Zecchini et al. (14)	melanoma	M	45	ipi + nivo	1	Unilateral facial palsy	normal	valacyclovir + prednisone	13 days	CR	Permanent discontinuation of ipi + continuation of nivo monotherapy	skin toxicity
Yuen C,et al (10).	melanoma	F	49	ipi + nivo	NM	Unilateral facial palsy	Linear enhancement of the facial nerves	Dexamethasone	1 weak	CR	NM	Autoimmune hemolytic anemia
	melanoma	M	68	ipi + pembro	NM	Unilateral facial palsy	Diffuse left facial nerve enhancement	Acyclovir	23 weeks	CR	NM	no
	melanoma	M	54	ipi	NM	Unilateral facial palsy	Enhancement of the right facial nerve	Prednisone + valacyclovir	4 weeks	CR	NM	Neuropathy (GBS like syndrome) + autoimmune thyroiditis
	bladder cancer	M	65	atezo	NM	Unilateral facial palsy	normal	Prednisone	3 weeks	CR		Polyradiculopathy + neuropathy
	melanoma	M	39	ipi+ nivo	NM	Unilateral facial palsy	Enhancement of the right facial, bilateral oculomotor and the V2 segments of trigeminal nerves	Prednisone + valacyclovir	8 weeks	PR	Permanent discontinuation of atezo	no
Bruno, et al. (11)	adenocarcinoma of the lung	M	72	pembro	3	bilateral facial palsy + bilateral ptosis and ophthalmoplegia + dysphonia + dysphagia.	normal	IV immunoglobulins		CR	NM	NM
Yost et al. (16).	melanoma	M	64	pembro	3 months after 14 months of ICI	Bilateral facial + diffuse areflexia + flaccid dysarthria (bifacial weakness variant of Guillain-Barre syndrome)	subtle enhancement of the facial nerves bilaterally	Intravenous immunoglobulin + prednisone	2 weeks		NM	Autoimmune hepatitis
Vogrig et al. (17).	kidney cancer	M	51	ipi + nivo	4	unilateral facial palsy	normal	corticosteroids	3 weeks	CR	discontinuation of ipi	NM
Beninato, et al. (18)	melanoma	M	48	ipi	NM	Unilateral facial palsy	NM	Prednisone	86 days	CR	Permanent discontinuation of ipi	no
Altman et al. (8)	melanoma	M	32	ipi + nivo	2	Unilateral facial palsy Bilateral facial palsy after C3 ipi+nivo	normal	Prednisone	8 weeks	PR	Permanent discontinuation of ipi after C3	no
Yan et al. (19)	NM	M	74	nivo	1	Unilateral facial palsy	NM	Oral steroid	NM	NM	NM	no
Takemura et al. (20)	kidney cancer	M	55	nivo	1	Unilateral facial palsy	enhancement of the left facial nerve	Prednisone	8 weeks	CR	Permanent discontinuation of nivo	no
Kichloo et al. (12)	small cell lung cancer	M	68	atezo	5	Unilateral facial palsy	normal	Prednisone	4 weeks	CR	Temporary discontinuation	no
Zieman et al. (21)	melanoma	F	51	ipi + nivo	1	Unilateral facial palsy	NM	Prednisone + valacyclovir	3 weeks	CR	Permanent discontinuation of ipi + continuation of nivo monotherapy	no
	melanoma	F	68	ipi + nivo	2	Unilateral facial palsy	NM	Prednisone + valacyclovir	3 weeks	CR	Permanent discontinuation of ipi + continuation of nivo monotherapy	Autoimmune hepatitis + autoimmune colitis
Numata et al. (22)	melanoma	M	46	ipi + nivo	1	Unilateral facial palsy	normal	hydrocortisone	2 weeks	PR	Temporary discontinuation of ipi	Bilateral anterior uveitis + vitiligo
Altman et al. (8)	melanoma	M	32	ipi + nivo	2	Bilateral facial palsy	NM	Prednisone	8 weeks	PR	Permanent discontinuation after 3 cycles of ipi	no

Ipi, ipilumumab; nivo, nivolumab; pembro, pembrolizumab; atezo, atezoluzumab; NM, not mentioned; CR, complete resolution; PR, Partial resolution; IV, Intravenous.

A systematic analysis published in 2023 assessed the risk of facial palsy associated with ICIs based on data from 21 randomized trials including 10,779 patients treated with ICIs (15). Patients were treated for several primary cancers: melanoma, gastro-esophageal cancer, renal or urothelial cancer and malignant mesothelioma. An increased risk of facial palsy associated with ICIs was reported in 23 cases with OR 3.07 (95% CI: 1.4–6.5) compared to control treatment. Results of subgroup analysis indicated that OR of ICI-related FP did not vary significantly by tumor type, ICIs treatment schedule, case of events, study design, median PFS and publication status. In the same analysis, the FDA Adverse Event Reporting System database was retrospectively reviewed between 2011 and 2022. In total, 274 cases were identified. Most patients presenting facial palsy were treated with ICI only (82.1%) rather than with combination of ICI with other drug (17.9%). The median onset time of facial palsy was 5.5 weeks in this review and 15 weeks in another case series (18). In most of the cases, drug interruption was performed (78%) and clinical outcome was favorable (71.7%).

### Neurological irAEs and ICI-induced facial palsy: epidemiology

Neurological irAEs (N-irAEs) are relatively rare with an estimated prevalence of 1-4% of ICI-treated patients (23, 24). Yet, with cardiological irAEs, neurological adverse events are among the most serious irAEs (25), with mortality rate of 28% for myasthenic syndrome and 21% for encephalitis. Thus, early detection, diagnosis and management of N-irAEs is a major issue. N-irAEs involving peripheral nervous system are twice more frequent than central nervous system (CNS) toxicity (7), and clinical as well as paraclinical characteristics of ICI-induced neuropathies differ from chemotherapy-induced neuropathies (26). Among N-irAEs, occurrence of facial palsy is particularly rare, with an incidence around 0.2% in the ICI-treated population (23 cases/10.779 patients) in the largest reported study (15). Furthermore, a recent study showed that combination of CTLA-4 and PD(L)-1 inhibitors is a risk factor of developing cranial neuropathies, including facial palsy (27).

Melanoma may have a potential risk of neurological irAE compared to other cancers. Indeed, in the literature described in **Table 1**, 12/18 patients presenting ICI-induced facial palsy are melanoma patients. This might be due to the early onset of ICI in melanoma patients since 2010 (which might increase the reported cases) and the high frequency of ICI combination, notably Ipi/Nivo which increase irAEs. However, a meta-analysis of 694 articles (7), melanoma was more frequent in patients with peripheral neuropathies (64/94, 68%; p = 0.003) and less common in encephalitis (19/56, 34%; p = 0.001).

### ICI-induced facial palsy: diagnosis

The diagnosis of facial palsy is primarily clinical. Physicians need to eliminate central facial paralysis that would lead to look for CNS involvement with simple neurological examination. Then, associated neurological symptoms, notably for immune-related myasthenia gravis, must be carefully searched to adapt patient’s further exams and surveillance (28). It is mandatory to exclude differential diagnoses such as stroke or toxic, metabolic, infectious and endocrine neuropathies. Nevertheless, the main goal is to eliminate a carcinologic progression such as brain or leptomeningeal metastases (17, 28). Differentiating immune-related from *a frigore* facial palsy (Bell’s palsy) can be difficult. The delay between ICI introduction and the occurrence of firsts symptoms is estimated to 2 months (0.5-17.0 months) and might help ICI imputability (17).

Even if some of them are missing in our report, biological exams are important to eliminate other etiologies of reversible neuropathies: HbA1c, vitamin B12, TSH, vitamin B6, folate, serum protein electrophoresis, immunofixation and CPK. Other exams might be done depending on the context: ANA, ESR, CRP, ANCA, anti–smooth muscle, SSA/SSB, RNP, anti-dsDNA, ganglioside antibodies, anti-MAG, anti-Hu (ANNA-1 ab), thiamine, Lyme, hepatitis B or C, and HIV. Thus, to our knowledge, anti-onconeural antibodies has always been reported as negative when reported in isolated ICI-related facial palsy (7, 10).

Lumbar puncture aims to seek for carcinomatous cells. CSF analysis may show a pleiocytosis (10, 17), but may also be normal (11, 14). Nerve conduction study shows a reduced amplitude in the facial nerve and an altered blink reflex (11). Brain MRI may reveal an ipsilateral facial nerve enhancement after gadolinium infusion, or be normal (10, 17).

In the case of our patient, due to the presence of hypophysitis, sarcoidosis diagnosis should be considered, but the patient did not present symptoms in the organs classically touched in this pathology (lungs, lymph nodes, joints, skin, eyes, heart, kidneys, etc.). Additionally, even if our patient did not complain of other symptoms and tendinous reflexes where present, electromyography could have been performed in this patient to increase diagnostic sensitivity (29).

### ICI-induced facial palsy: clinical management ant treatment

N-irAEs management first relies on ICI discontinuation. In some reported cases of combined ICI treatment, only one of them was discontinued (17). Oral corticosteroids (1-2 mg/kg/d) may be introduced, and slowly tapered within 4 to 6 weeks (27). Some authors have used intravenous immunoglobulin (IVIG) instead or with corticosteroids (17). It is mandatory to assess the disease course with early and repeated neurological examination to adapt patient’s management. In case of facial palsy being the inaugural symptom of a Guillain-Barre syndrome or a more diffuse neuropathy, further therapies might be considered such as plasma exchange (27).

The prognosis of isolated facial palsy is good with 70% of complete recovery, within a median of 41 days (18). However, recovery can be partial with persistent sequelae in approximately one patient out of four (17). In this context, rehabilitation strategies for facial nerve injuries is crucial and several techniques are available, including exercise, electrical stimulation, biofeedback, and facial neuromuscular retraining (30). In case of full recovery, reintroduction of ICI might be considered in case of absence of alternative therapy. Indeed, disease progression has been shown to be the main cause of death in patients with irAEs (24). To switch ICI subtype (from CTLA-4 to PD-1 inhibitors) in non-neurological irAEs has been associated to a lower risk of relapse (31), but evidence are lacking in N-irAEs. Yet, no clear guideline is currently available, and this attitude should be a case-by-case evaluation.

### Oncological perspective: quick and multi-disciplinary management

In conclusion, ICI-induced facial palsy is a rare complication that a medical oncologist rarely sees in his routine practice. The key in the management of these rare N-irAEs is the multi-disciplinary network between oncologists and neurologists or internists (32). This essential collaboration can take various forms, such as external consultants, immuno-toxicity meetings, mailing lists, messaging applications (33). Quick clinical evaluation, quick imaging and lab tests are essential to eliminate differential diagnoses and start early corticosteroid treatment, effective in 70-80% of cases (15, 34). Building a strong network to deal with rare and life-threatening irAEs is one of the new challenge of medical oncology, a medical specialty where multi-disciplinary care has always been highly estimated.

## Data availability statement

The original contributions presented in the study are included in the article/supplementary material. Further inquiries can be directed to the corresponding author.

## Ethics statement

The studies involving humans were approved by Institut Paoli Calmettes institutional review board. The studies were conducted in accordance with the local legislation and institutional requirements. The participants provided their written informed consent to participate in this study. Written informed consent was obtained from the individual(s) for the publication of any potentially identifiable images or data included in this article.

## Author contributions

EM: Writing – review & editing, Writing – original draft, Formal analysis, Data curation. GC: Writing – review & editing, Writing – original draft. RM: Writing – review & editing, Investigation. SC: Writing – review & editing, Visualization. NC: Writing – review & editing, Visualization. BC: Writing – review & editing, Supervision. AC: Writing – review & editing, Supervision, Conceptualization. PR: Writing – review & editing, Writing – original draft, Supervision, Methodology, Investigation, Data curation, Conceptualization.
